# Change of Management

**Published:** 1894-09

**Authors:** 


CHANGE OF MANAGEMENT.

The editors of THE JOURNAL OF COMPARATIVE MEDICINE AND VETERINARY ARCHIVES regret extremely the lapse of issue for several months, which has been caused by unusual demands upon their personal time. While those who have paid their subscriptions in advance have a right to feel aggrieved, as has been expressed in a number of communications, yet the editors have cause for grievance in the dilatory payments made by a majority of the subscribers. As shown in our editorial two years ago, the JOURNAL has been conducted, for the interest of the veterinary profession, at personal expense to the editors. The JOURNAL has had no school to advertise, or any secondary or ulterior motive to gain.

It has been conducted because its editors have believed that they were doing good, in furnishing the profession with a liberal magazine, free to the pen of all. It became too great a drain on the time and pockets of two men, but they were unwilling to let it die.

Dr. W. A. Conklin and Dr. R. S. Huidekoper have, therefore the pleasure to announce that they have associated with Dr. W. Horace Hoskins, of Philadelphia, and Dr. A. W. Clement, of Baltimore. Both of these gentlemen are too well known as scientific and ardent workers in the cause of veterinary medicine to need eulogy.

The business management of the JOURNAL is in the hands of

DR. W. HORACE HOSKINS,
3452 Ludlow St., Philadelphia, Penn., where all payments must be made, and all matters addressed concerning subscriptions, advertisements, or other business matters. Reading matter, original articles, and other communications, will still be addressed to

DR. R. S. HUIDEKOPER,
155 W. 56th St., New York City,

Or to DR. A. W. CLEMENT,
916 Cathedral St., Baltimore, Md.

